# Increased mortality rates in young and middle-aged patients with malignant germ cell tumours

**DOI:** 10.1038/sj.bjc.6601558

**Published:** 2004-02-03

**Authors:** S D Fosså, N Aass, S Harvei, S Tretli

**Affiliations:** 1 The Norwegian Radium Hospital, Department of Clinical Research, Montebello, N-0310 Oslo, Norway; 2 The Cancer Registry of Norway, Montebello, N-0310 Oslo, Norway

**Keywords:** malignant germ cell tumours, mortality, nongerm cell cancer, cardiovascular diseases

## Abstract

Cisplatin-based chemotherapy of malignant germ cell tumours (MGCT) has been reported to increase the risk of cardiovascular morbidity. A high incidence of second nongerm cell malignancies is well documented in MGCT survivors. The death risk due to these conditions is, however, more unknown in MGCT patients. Standard mortality rates (SMRs) were established in 3378 Norwegian MGCT patients treated from 1962 to 1997 aged ⩽55 years. The patients represented three principal treatment strategies: 1962/1969 (period 1): radiotherapy only; 1970/1979 (period 2): radiotherapy with or without noncisplatin-containing chemotherapy; 1980/1997 (period 3): surgery only or radiotherapy or cisplatin-based chemotherapy. Patients were censored when they reached the age of 60 years. Patients not dying from MGCT displayed significantly increased SMRs for respectively diseases of the circulatory system (SMR: 1.2, 95% confidence interval (CI): 1.0–1.5), benign gastrointestinal disorders (SMR: 2.1, 95% CI: 1.1–3.5) and nongerm cell malignancies (SMR: 2.0, 95% CI: 1.7–2.4). The SMRs for diseases of the circulatory system were similar in the three observation periods, whereas the highest SMR for benign gastrointestinal disorders was observed in patients from period 2. The risk of dying from a nongerm cell malignancy was increased both in periods 2 and 3. In conclusion, although the overall SMR for diseases of the circulatory system is increased in MCGT survivors, the introduction of cisplatin-based chemotherapy into the treatment of MGCT has so far not resulted in increased death rates due to these conditions. Patients with MGCT have a significantly increased relative death risk due to a second nongerm cell cancer, even after the introduction of modern treatment principles with overall reduction of radiotherapy. The increased death risk due to benign gastrointestinal disorders, probably related to radiotherapy, requires future in-depth analysis.

Malignant germ cell tumours (MGCT), about 95% of them being testicular cancer, represent the most frequent malignancy in men aged 20–34 years (Engeland *et al*, 1993), and the incidence is rising. For many years radiotherapy has been the cornerstone in the treatment of testicular seminoma, and ⩾98% of the patients become long-term survivors (Fosså *et al*, 1999). Although the surveillance policy has been introduced into the treatment of seminoma (Warde *et al*, 2002), adjuvant radiotherapy will world-wide continue to be the standard treatment in many countries where adequate and frequent follow-up of the patients is not feasible. After the introduction of cisplatin-based therapy in the 1970s, about 90% of the patients with nonseminoma are cured. This implies that most patients with MGCT theoretically have a life expectancy of 30–50 years after their successful treatment. It is, however, known from single institution studies and from large cancer registry-based reports that testicular cancer survivors have an increased incidence of second cancer (van Leeuwen *et al*, 1993; Hoff Wanderås *et al*, 1997; Travis *et al*, 1997, 2000). The increase has been most pronounced for sarcoma, malignancies of the gastrointestinal tract, lung cancer and for bladder cancer. These solid cancers develop mostly 10 years or more after radiotherapy, when patients are no longer followed up intensively by their primarily responsible oncological unit. Epidemiologically less established are observations on late adverse events related to obesity, hypercholesterolaemia and decreased kidney function and to cardiovascular events (Hansen *et al*, 1988; Ellis *et al*, 1992; Raghavan *et al*, 1992; Bokemeyer *et al*, 1996; Fosså *et al*, 1989; Meinardi *et al*, 2000; Huddart *et al*, 2003; Fosså *et al*, 2003; Nord *et al*, 2003a). Single-centre reports have indicated that such conditions may be associated with the modern treatment of MGCT, in particular with the use of cisplatin-based chemotherapy. Late cardiac complications are, however, also described after mediastinal irradiation (Lederman *et al*, 1987), a common adjuvant treatment strategy before 1980 in seminoma patients. Furthermore, the possibility exists that the development of cardiovascular diseases in patients with MGCT is related to the disease itself, for example mediated by the relative hypogonadism, observed in 10–15% of the patients (Petersen *et al*, 1998; Nord *et al*, 2003b).

There is no doubt that moderate dosed radiotherapy as applied today in MGCT may lead to a slight degree of gastrointestinal dysfunction and peptic ulcers in 3–10% of the cases (Fosså *et al*, 1989; Yeoh *et al*, 1995). The occurrence of more severe gastrointestinal disorders is rare, but severe and even lethal complications have been described many years after the combination of radiotherapy and chemotherapy. This combination is still valid for irradiated patients with seminoma, developing a relapse.

Although the incidence of second malignancies (van Leeuwen *et al*, 1993; Hoff Wanderås *et al*, 1997; Travis *et al*, 1997, 2000) in relation to the patient's primary treatment and the occurrence of gastrointestinal of cardiovascular disorders (Fosså *et al*, 1989; Yeoh *et al*, 1995) after chemotherapy have been described in several reports, much less is known about the *causes of death* in patients who are successfully treated for MGCT. Large epidemiological studies are lacking, which prove any relation between different treatment modalities of MGCT and late nonmalignant complications. One reason for the lack of such large-scale data is the fact that only few countries have population-based registries for gastrointestinal or cardiovascular disorders, which can be used for comparison. In this situation, the analysis of mortality data may increase the understanding of any relation between the treatment of MGCT and the development of subsequent lethal, but benign disorders. Although such an analysis *per se* must be retrospective and is largely based on treatments no longer relevant for MGCT, many patients with MGCT treated according to ‘old-fashioned’ strategies are still alive and at risk to develop serious complications often 15–20 years after their treatment. Furthermore, the results obtained in patients with MGCT as to late treatment-related mortality may be relevant for the oncological therapy of solid malignancies in general.

From a clinical point of view, one would expect increased mortality rates due to second nongerm cell malignancies, as most of these are cancer types with a generally poor prognosis. It is, however, more uncertain as to whether a high incidence of cardiovascular morbidity among MGCT patients, if at all present, corresponds with a raised mortality rates due to diseases of the circulatory system. With this background, the primary aim of the present study is to present mortality rates for diseases of the circulatory system among MGCT survivors compared with comparable rates in the age-matched male population. We also include the analysis of death rates from nongerm cell cancer, and from selected nonmalignant diseases among the same patients.

## PATIENTS AND METHODS

Cancer reporting to the Cancer Registry of Norway (NCR) has been compulsory since the registration started in 1953. All hospitals and histopathological laboratories are committed independently to report all new cases of cancer, also subsequent primary cancers, including information at the time of diagnosis and extent of the disease. The latter is categorised as locally confined, regionally advanced or distant metastatic. The registration of basal cell carcinoma has not been quality-secured, and these cancer cases are excluded from the present analysis. All death certificates are coded by Statistics Norway and linked to the NCR through the unique personal identification number given to every citizen in Norway. This linkage provides the information on the date and cause of death. All patients were followed up with respect to cause of death until the end of 1997.

The NCR is incomplete as to the primary treatment of cancer patients and their clinical follow-up. Therefore, the patients were grouped into three cohorts based on the year of diagnosis of MGCT:

NCR-1: diagnosed between 1962 and 1969 (period 1)
NCR-2: diagnosed between 1970 and 1979 (period 2)
NCR-3: diagnosed between 1980 and 1997 (period 3)

These time intervals mirror major changes in the postorchiectomy treatment principles of Norwegian TC patients: nearly all patients from the NCR-1 group were treated with high-voltage radiotherapy alone. Nonmetastatic patients with seminoma or nonseminoma underwent infradiaphragmatic radiotherapy, respectively, 40 and 50 Gy, whereas patients with metastases had mediastinal irradiation and/or palliative limited field radiotherapy. Chemotherapy was not used in general, and, if given, cyclophosphamide was the only available drug before 1971. Nonmetastatic patients from the NCR-2 group were treated similarly to those from the NCR-1 group, while metastatic patients received available chemotherapy (cyclophosphamide, mitomycin, actinomycin D, adriamycin, bleomycin, vinblastine, metothrexate) in combination with abdominal and mediastinal irradiation. From 1980 (NCR-3 group), cisplatin-based chemotherapy represented the standard treatment for patients with metastatic nonseminomatous MGCT. Nonmetastatic patients with nonseminoma underwent retroperitoneal lymph node dissection (RPLND) or were included into a surveillance programme (from 1989). Adjuvant abdominal radiotherapy was restricted to seminoma patients without metastases, the target dose being ⩽30 Gy in most patients. The use of prophylactic mediastinal irradiation was discontinued. During the early 1980s, patients with metastatic seminoma received postchemotherapy radiotherapy to residual masses. This policy was gradually abandoned after 1985.

Of all TC patients, 9% were older than 55 years of age at the time of diagnosis. These patients were excluded from all analyses to avoid a group of men having a considerable risk of diseases of the circulatory system, *a priori* probably not related to their TC. Due to this high incidence of circulatory disorders in elderly men of the general population, any existing but presumably small difference would remain undetectable. Due to the same reason, patients were censored in the analysis when they reached the age of 60 years (all together 122 patients).

The observed number of deaths from a specified condition was compared with the expected number of deaths estimated by applying the specific mortality rates for 1-year age groups and 1-year birth years in the MGCT-patient group under consideration. Based on the observed (OBS) and expected (EXP) numbers of deaths, standardised mortality ratios (SMRs) were calculated. The absolute excess risk (AER) is calculated by the difference between the observed and expected numbers of deaths divided by the number of person years and multiplied by 10 000. The estimated 95% confidence intervals (95% CI) for both SMR and AER were based on the assumption that the observed numbers were Poisson distributed.

## RESULTS

**Figure 1:**
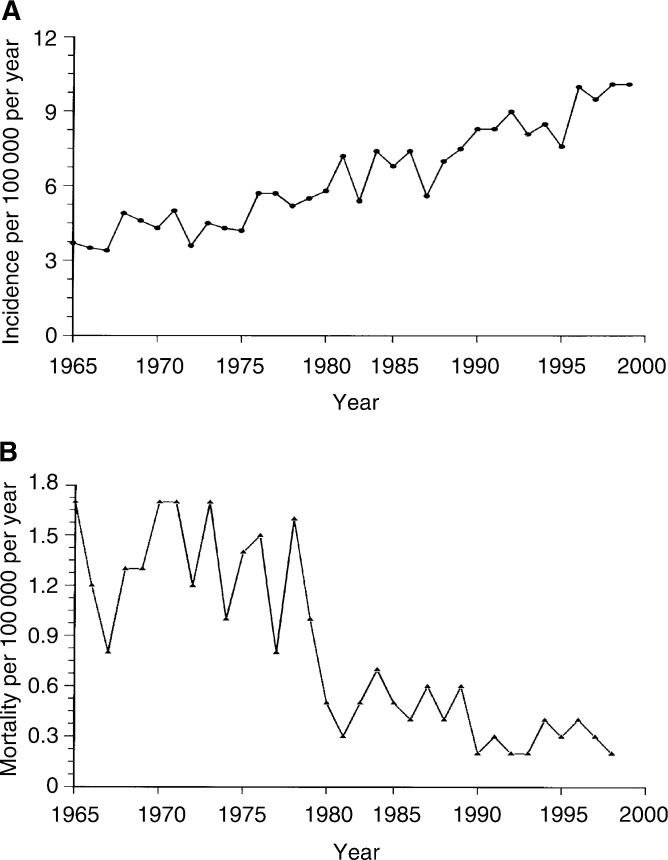
Age-adjusted (Based on World Standard population) incidence (**A**) and mortality (**B**) rates for patients with malignant germ cell tumours diagnosed from 1965 to 1999 (The Cancer Registry of Norway, personal communication, 2002).

The series comprises 3378 patients representing 41 960 patients years. The distribution of seminoma *vs* nonseminoma was about 1 : 1. Distribution of the extent of the disease at the time of diagnosis was similar in the three groups. In spite of an increasing incidence of MGCT, the overall mortality in these patients (all causes included) has decreased dramatically after 1980, when cisplatin became available for the treatment of Norwegian TC patients (Figure 1

).

The main cause of death was, of course, MGCT with 493 out of 609 cancer deaths. A total of 116 patients died from other types of cancer, which gave an SMR of 2.0 (95% CI: 1.7–2.4) (Table 1

**Table 1:** Standard mortality ratio and absolute excess risk (per 10 000 person years) for patients with malignant germ cell tumours (The Norwegian Cancer Registry, 1962–1993)

	**ICD-9**		**NCR-1 1962–1969**	**NCR-2 1970–1979**	**NCR-3 1980–1997**	**Total 1962–1997**
# of patients			570	870	1938	3378
# Person years			9330	12 768	19 862	41 960
Disease of the circulatory system	390–458	OBS	50	40	17	107
		EXP	42.5	31	14	88.4
		SMR	1.2 (0.9, 1.6)^a^	1.3 (0.9, 1.8)	1.1 (0.7, 1.8)	1.2 (1.0, 1.5)
		AER	8.0 (−5.7, 24.5)	7.0 (−1.9, 18.4)	1.5 (−2.1, 6.6)	4.5 (−0.4, 9.3)
Myocardial infarction	410–414	OBS	35	20	14	69
		EXP	29.6	22.1	10.5	62.2
		SMR	1.2 (0.8, 1.6)	0.9 (0.6, 1.4)	1.3 (0.7, 2.2)	1.1 (0.9, 1.4)
		AER	5.7 (−5.5, 20.5)	−1.6 (−7.8, 6.8)	1.8 (−1.4, 6.6)	1.6 (−2.0, 6.0)
Gastrointestinal disorders	520–577	OBS	3	7	4	14
		EXP	2.6	2.4	1.8	6.7
		SMR	1.2 (0.2, 3.4)	3.0 (1.2, 6.1)	2.2 (0.6, 5.8)	2.1 (1.1, 3.5)
		AER	0.4 (−2.2, 6.6)	3.6 (0.3, 9.4)	1.1 (−0.4, 4.3)	1.8 (0.2, 4.0)
Accidents, poisoning, suicide	800–999	OBS	11	9	14	34
		EXP	10.1	12.7	17.3	40.1
		SMR	1.1 (0.5, 2.0)	0.7 (0.3, 1.3)	0.8 (0.4, 1.4)	0.8 (0.6, 1.2)
		AER	1.0 (−5.0, 10.3)	−2.9 (−6.8, 3.5)	1.7 (−4.8, 3.1)	−1.4 (−3.9, 1.8)
Nongerm cell cancer	140–239 minus 186	OBS	32	56	28	116
		EXP	24.9	20.2	11.8	56.9
		SMR	1.3 (0.9, 1.8)	2.8 (2.1, 3.6)	2.4 (1.6, 3.4)	2.0 (1.7, 2.4)
		AER	7.6 (−3.4, 21.8)	28.0 (17.3, 41.1)	8.2 (3.4, 14.4)	14.1 (9.0, 19.1)
Stomach cancer	151	OBS	2	9	2	13
		EXP	2.1	1.5	0.8	4.4
		SMR	1.0 (0.1, 3.4)	5.9 (2.7, 11.3)	2.5 (0.3, 9.1)	3.0 (1.6, 5.0)
		AER	−0.1 (−2.1, 5.5)	5.8 (2.00, 12.2)	0.6 (−0.3, 3.3)	2.1 (0.6, 4.2)
Pancreatic cancer	157	OBS	4	3	2	9
		EXP	1.4	1.1	0.6	3.1
		SMR	2.8 (0.8, 7.1)	2.7 (0.6, 7.8)	3.3 (0.4, 11.9)	2.9 (1., 5.4)
		AER	2.8 (−0.3, 9.4)	1.5 (−0.4, 6.0)	0.7 (−0.2, 3.3)	1.4 (0.2, 3.4)
Lung cancer	162	OBS	7	11	6	24
		EXP	6.0	5.0	2.6	14
		SMR	1.2 (0.5, 2.4)	2.2 (1.1, 4.0)	2.3 (0.8, 4.9)	1.7 (1.1, 2.6)
		AER	1.1 (−3.5, 9.0)	4.7 (0.4, 11.5)	2.0 (−0.2, 5.3)	2.3 (0.3, 5.2)

OBS=observed number; EXP=expected number; SMR=standardiszed mortality ratio; AER=absolute excess risk.

a 95% confidence interval.

). The relative risk rates of dying from nongerm cell cancer were lowest during period 1 with significantly raised SMRs in patients treated during periods 2 and 3. Standard mortality rates were significantly increased for stomach, pancreatic and lung cancer, in particular in patients from the NCR-2 group. These relative risk rates are associated with significantly raised figures of AER.

A total of 181 TC patients died from benign conditions, while 163 deaths were expected (SMR: 1.1; 95% CI: 0.95–1.27). All patients combined displayed a slight, although significantly increased relative risk of dying from diseases of the circulatory system (ICD-9: 390–458), the SMR being 1.2 (95% CI: 1.0–1.5), with a nonsignificantly increased risk of dying from myocardial infarction (ICD-9: 410–414), the SMR being 1.1 (95% CI: 0.9–1.4) (Table 1). Within each of the three diagnostic periods separately, the risk of dying from circulatory disorders was, however, not significantly increased. Neither was there a change of these mortality rates comparing the three time periods with each other. Notably, the relative risk of dying from diseases of the circulatory system has so far remained unchanged after the introduction of cisplatin-based chemotherapy into the treatment of MGCT. Neither do the figures for AER mirror an increased risk of cardiovascular death during the third period, although the respective CIs were wide and overlapping, without reaching the level of statistical significance.

The SMR and AER for benign gastrointestinal disorders (ICD-9: 520–577) were significantly increased for the three diagnostic periods combined with a particular increase within the NCR-2 group when the combination of chemotherapy containing adriamycin and actinomycin D, with radiotherapy had gained some popularity. The 14 deaths were due to liver cirrhosis or unspecified liver diseases (six patients), cholelithiasis (two patients), acute vascular disorders of the gastrointestinal tractus (two patients), oesophago-gastro-duodenal ulcus (two patients), peritonitis (one patient) and appendicitis (one patient). All but two of these patients had had infra-diaphragmatic radiotherapy from 6 to 22 years prior to death. In all, 34 MGCT patients died from accidents, poisoning or suicide. This number did not significantly deviate from 40.1 cases expected in the general population.

## DISCUSSION

This report documents an increased risk for cured MGCT patients to die from nongerm cell cancer, from diseases of the circulatory system and from benign gastrointestinal disorders. No obvious differences as to the period of treatment were observed for the risk of dying from circulatory disorders. The combination of radiotherapy with noncisplatin-containing chemotherapy (NCR-2 group) represented a particularly high risk of dying from benign gastrointestinal disorders. As expected, the increasing use of cytostatics, especially if combined with radiotherapy (period 2), increased the death rate due to a second nongerm cell cancer.

Based on long-term studies, often performed in more limited series of testicular cancer survivors, several investigators have reported an increased prevalence of cardiovascular risk factors and disorders of the circulatory system in MGCT patients after curative cisplatin-based chemotherapy (Bokemeyer *et al*, 1996; Meinardi *et al*, 2000; Huddart *et al*, 2003). After such cytostatic treatment, patients more often had increased serum levels of cholesterol, weight gain, hypertension and decreased renal function (Hansen *et al*, 1988; Ellis *et al*, 1992; Raghavan *et al*, 1992; Bokemeyer *et al*, 1996; Nord *et al*, 2003a) than the age-matched general population, or compared to MGCT patients treated by surgery only. Our data support the view of increase of diseases of the circulatory system in patients with MGCT, as our complete cohort of MGCT patients indeed displayed a marginally increased risk of dying from circulatory diseases (SMR: 1.2 (95% CI: 1.0–1.5)). Based on the above literature, we had expected particularly increased mortality rates due to cardiovascular disorders after the introduction of cisplatin-based chemotherapy in 1980 (NCR-3 group), but so far we have been unable to document such figures. If one accepts the association between the year of diagnosis and treatment, as described in the section of Patients and methods, and the limitation of a short follow-up period for the third group, the death risk due to circulatory disorders, including myocardial infarction, was not related to the use of cisplatin-based chemotherapy . No increased risk was seen in the patients from the NCR-3 group; about 40% of them had been treated with cisplatin-based chemotherapy and only exceptionally combined with radiotherapy. Several reasons may explain the lack of the expected elevated postchemotherapy risk of cardiovascular deaths in the third period. Cardiac irradiation in patients without chemotherapy may be a confounding factor, reducing any existing intergroup difference: even after 1980, the routine target radiation field in patients with early testicular cancer includes the lower part of the heart, generally resulting in radiation doses of 30–90 cgy of the whole heart or parts of it (Huddart *et al*, 2003). Furthermore, during the first two periods of our study, patients with lymph node metastases had mediastinal radiotherapy at doses of 40–45 Gy, including major parts of the heart. From the experience in patients with Hodgkin's disease (Glanzmann *et al*, 1998), it is known that mediastinal radiotherapy increases the risk of subsequent cardiomyopathy. Furthermore, older drugs used in the treatment of advanced testicular cancer before 1980, such as Actinomycin D or Adriamycin may have enhanced such radiation effect or are cardiotoxic by themselves.

Our data on mortality rates due to circulatory disorders do not, however, completely exclude the possibility of an increased risk of dying from cardiovascular diseases related in particular to cisplatin-based chemotherapy of MGCT, as our follow-up ends in 1997, only 17 years after the introduction of cisplatin in the treatment of MGCT. Longer follow-up may change the shown pattern of mortality risks. We can neither exclude the possibility that cisplatin-based chemotherapy is related to an increased risk of cardiovascular morbidity. During the 1980s and 1990s, improved treatment of hypertension and hyperlipidaemia and more successful management of myocardial infarction may have led to reduced mortality rates due to circulatory diseases, in spite of persistent or even increasing morbidity rates (Molstad and Andersen, 2002). Regrettably, no Norwegian national population-based registries are available on diseases of the circulatory system, which could prove this hypothesis of increasing morbidity and decreasing mortality.

One should also discuss alternative and treatment-independent causes leading to cardiovascular events and high-risk factors in testicular cancer survivors. Slight hypogonadism as reduced testosterone serum levels and/or increased LH serum levels are seen in about 15% of these patients (Huddart *et al*, 2003; Nord *et al*, 2003a). Hypogonadism may by itself lead to premature ageing with its consequences on disorders of the circulatory system, including increased BMI, serum lipid disturbances and hypertension. Future comparative long-term studies in patients treated after 1980 and following modern treatment strategies have to further clarify the impact of cisplatin-based chemotherapy or of other factors on the development of cardiovascular disorders.

A completely unexpected observation was the significantly raised death rate due to benign gastrointestinal disorders. Abdominal radiotherapy at doses of 40–50 Gy, in particular if combined with chemotherapy seems to contribute considerably to this risk, especially in patients treated before cisplatin became available in Norway (period 2). This is in agreement with our previous observations of increased gastrointestinal morbidity after radiotherapy alone or after the combination of abdominal radiotherapy with adriamycin-containing chemotherapy (Fosså *et al*, 1989; Hoff Wanderås *et al*, 1994). Our findings also show that gastro-duodenal ulcer, recognised as radiotherapy-induced long-term toxicity (Fosså *et al*, 1989), not only represents a significant morbidity but may also be the patient's cause of death. This observation is in contrast to Horwich and Bell's (1994) report, who did not find an excess of the death rate of benign disorders after radiotherapy for seminoma. The high SMR due to disorders of the liver or its excretory duct system was surprising and requires a future in-depth analysis. At the present time, we propose the following explanation: Fibrosis is a well-known dose-dependent late side effect of radiotherapy, although its development only rarely causes clinical symptoms in testicular cancer survivors. However, retroperitoneal fibrosis may exceptionally cause stenoses of the ureteres and/or of the bile or hepatic ducts (Moul, 1992; Biermann *et al*, 1994) 6–20 years after radiotherapy. The fibrotic changes in the upper retroperitoneal space may even mimic pancreatic cancer (Stensvold *et al*, 2003). In rats, whole-liver radiation with 24 Gy resulted in significant liver fibrosis that predicts the onset of liver dysfunction, the process being enhanced by cyclophosphamide (Geraci *et al*, 1992). In humans, 30 Gy can safely be given to the liver, and even higher radiation doses are tolerated, if only parts of the liver are irradiated (Dawson *et al*, 2001). However, as in the animal experiments, cytostatic drugs may act as radiosensitisers. The relatively high percentage of causes of death related to the liver or its excretory system (eight of 14) may thus be a consequence of irradiation-induced fibrotic changes in the upper retroperitoneal space. The two deaths due to cholelithiasis (according to the certificate of death) may prove to represent erroneous diagnoses, established at times when computer tomography (CT) was not available. Computer tomography would possibly have shown fibrotic changes in the upper abdomen, leading to chronic stenosis of the bile duct system. Whether the mortality rate of benign gastrointestinal complication is reduced by modern radiotherapy (reduced target doses, more limited fields) has to be proven by forthcoming analyses.

As expected, the overall mortality rate due to second nongerm cell cancer was high, in patients from the NCR-2 group, whereas radiotherapy alone (NCR-1 group) was not followed by an increased SMR for second cancer. The significantly increased SMR of 2.4 in NCR-3 group may in part be due to the continuous use of combined chemo-radiotherapy in some patients with advanced seminoma, a strategy that was first discontinued from 1985 onwards. At present, the continuously increased SMR due to second cancer after 1980 in spite of simultaneous reduction of radiotherapy warrants the oncologist's awareness and some reluctance to use chemotherapy if not absolutely necessary for cure. It is well known that high-dose cisplatin-based chemotherapy may increase the risk of secondary leukaemia (Travis *et al*, 2000). However, all 28 deaths in the NCR-3 group were due to solid malignancies. This observation warrants that patients cured from MGCT by chemotherapy, especially if they also have received radiotherapy, should have a life-long follow-up with the aim to diagnose a second cancer as early as possible. Furthermore, the risk of a second cancer should always be considered if an individual long-term survivor after successful treatment of MGCT experiences new and unexpected health problems.

One important weakness of our study has to be discussed: We have no individual treatment data as the information on primary and, in particular, secondary therapy of MGCT is incomplete in the NCR. The allocation to time periods as done in the present paper yields the best surrogate estimation of treatment of MGCT available for us in this population-based series.

In summary, MGCT survivors have a significantly increased risk of dying from diseases of the circulatory system, benign gastrointestinal disorders and second nongerm cell cancer. After the introduction of cisplatin into the treatment of MGCT, the death rate from diseases of the circulatory disease has, however, not increased as compared to the preceding decade, when metastatic patients had been treated with extended radiotherapy, increasingly combined with noncisplatin containing radiotherapy. Radiotherapy, in particular, if combined with chemotherapy, generally seems to increase the death rate of second nongerm cell cancer and also seems to contribute to the death rate from benign gastrointestinal disorders.

Further comparative studies in patients treated by either surgery alone, radiotherapy alone or chemotherapy are urgently needed to define the risk of serious long-term toxicity after modern treatment of patients with MGCT.
